# Absence of Protoheme IX Farnesyltransferase CtaB Causes Virulence Attenuation but Enhances Pigment Production and Persister Survival in MRSA

**DOI:** 10.3389/fmicb.2016.01625

**Published:** 2016-10-24

**Authors:** Tao Xu, Jian Han, Jia Zhang, Jiazhen Chen, Nan Wu, Wenhong Zhang, Ying Zhang

**Affiliations:** ^1^Key Laboratory of Medical Molecular Virology, Huashan Hospital, Shanghai Medical College of Fudan UniversityShanghai, China; ^2^Department of Pathogenic Biology, School of Basic Medical Sciences, Lanzhou UniversityLanzhou, China; ^3^Department of Molecular Microbiology and Immunology, Bloomberg School of Public Health, Johns Hopkins UniversityBaltimore, MD, USA

**Keywords:** *Staphylococcus aureus*, heme, antibiotics, pigment, virulence, persister formation

## Abstract

The membrane protein CtaB in *S. aureus* is a protoheme IX farnesyltransferase involved in the synthesis of the heme containing terminal oxidases of bacterial respiratory chain. In this study, to assess the role of CtaB in *S. aureus* virulence, pigment production, and persister formation, we constructed a *ctaB* mutant in the methicillin-resistant *Staphylococcus aureus* (MRSA) strain USA500. We found that deletion of *ctaB* attenuated growth and virulence in mice but enhanced pigment production and formation of quinolone tolerant persister cells in stationary phase. RNA-seq analysis showed that deletion of *ctaB* caused decreased transcription of several virulence genes including RNAIII which is consistent with its virulence attenuation. In addition, transcription of 20 ribosomal genes and 24 genes involved in amino acid biosynthesis was significantly down-regulated in the *ctaB* knockout mutant compared with the parent strain. These findings suggest the importance of heme biosynthesis in virulence and persister formation of *S. aureus*.

## Introduction

*Staphylococcus aureus*, named according to production of golden pigment, is an important human pathogen causing a variety of infection types including rampant skin and soft tissue infections, pneumonia, septicaemia, endocarditis, and central nervous system (CNS) infections. Methicillin-resistant *S. aureus* (MRSA) is notorious for its development of antibiotic resistance and expression of multiple virulence factors. (Li et al., 2012; Carrel et al., 2015).

*S. aureus* virulence factors are multifactorial and previous studies have been mainly focused on toxins (α-toxin, γ-toxin, Panton-Valentine leucocidin, exfoliative toxin and phenol-soluble modulins, etc.), surface proteins (FnbP, Bap, SasX, etc.) that help bind to host cells, facilitate internalization and immune evasion. Staphyloxanthin, synthesized from farnesyl diphosphate (FPP) by CrtM and CrtN, is the main component of *S. aureus* golden pigment (Liu et al., 2005). Staphyloxanthin not only plays a protecting role in bacterial fitness, but enhances virulence and survive attack by neutrophils (Clauditz et al., 2006). In addition, global regulatory systems (Agr, SaeRS, SarA, etc.) govern different aspects of physiology and expression of virulence traits, maintaining a balance between fitness and virulence.

It was in *staphylococcus* that persisters were first described in Bigger (1944). Persisters represent a certain portion of a bacterial culture that is genetically identical but phenotypically resistant or tolerant to antibiotics and stresses. In the model organism *Escherichia coli*, much research reveals the mechanisms of persister formation, including involvement of toxin-antitoxin, protein degradation, energy production and DNA repair (Zhang, 2014). However, less understood are the mechanisms of *S. aureus* persister formation. The portion of persisters in *S. aureus* is so high that a hypothesis was proposed that unlike *E. coli*, all *S. aureus* cells in stationary phase are persisters (Keren et al., 2004). Subsequently, however, Lechner et al. proved that stationary phase cultures of *S. aureus* are also a mixture of regular and persister cells (Lechner et al., 2012).

Though the key mechanisms of *S. aureus* persister formation are poorly understood, progress has been made recently. It has been reported that biofilm formation (Lewis, 2001; Resch et al., 2006) and small colony variants (SCV; Lechner et al., 2012) are two key features involving *S. aureus* persister formation, probably because the cells in biofilms and SCV cells have a different profile of gene expression, which makes them more readily to form persisters. Glycerol uptake has been reported to play a role in persister formation. Mutation in the glycerol transporter encoding gene *glpF* caused defective survival of *S. aureus* to ampicillin and norfloxacin (Han et al., 2014). A point mutation of the inorganic phosphate transporter gene *pitA* enhanced tolerance to daptomycin (Mechler et al., 2015). Mutations in purine biosynthesis genes (*purB, purF, purH, purM*,) amino acid, lipid, carbohydrate metabolism, and energy production genes efflux etc. were found to cause decreased persister formation in recent transposon mutant library screens (Yee et al., 2015; Wang et al., 2015).

Heme synthesis is an important pathway in Gram positive bacteria and provide substrate to production of terminal oxidases (Mogi et al., 1994). Within vertebrates *S. aureus* fulfills its requirement of iron by uptaking heme-iron from transferrin or heme or hemoglobin with its several transporters including StrA, StrB, IsdA, and IsdE, etc. (Drabkin, 1951; Mazmanian et al., 2003; Liu et al., 2008; Mason and Skaar, 2009). However, in an environment without heme-iron, *S. aureus* has to synthesize heme A with a complex pathway starting from glutamate (Hammer et al., 2016). CtaB and CtaA catalyzes the last two steps of the process. CtaB is a heme O synthase (protoheme IX farnesyltransferase) and while CtaA is an integral membrane protein that converts heme O to heme A (Svensson et al., 1993; Svensson and Hederstedt, 1994; Clements et al., 1999). Heme A is essential for functional expression of the terminal oxidases. Among terminal oxidases synthesized with heme A, cytochrome aa 3 are quinol oxidases (QoxA, QoxB, etc.) and cytochrome caa 3 is a cytochrome c oxidase.

Though heme synthesis mainly contributes to the pathway of synthesis of terminal oxidases that mediate bacterial respiration, it has also been reported to participate in fitness and virulence of *S. aureus*. For example, CtaA was found to be required for starvation survival and recovery from glucose starvation (Clements et al., 1999). A correlation between heme production and pigment production was reported by Lan et al., as depletion of CtaA and QoxB both enhanced pigment production, while attenuating hemolytic activity and virulence (Lan et al., 2010). However, no study has been done to address the specific effects of *ctaB* mutation on the heme-to-respiratory chain pathway and associated phenotypic changes. In this study, we created a CtaB deletion mutant of *S. aureus* and found associations of CtaB with heme synthesis, pigment production as well as persister cell formation. In addition, we performed a transcriptome analysis to provide new insights into the basis of the above associations.

## Material and methods

### Bacterial strains, growth, and chemical reagents

*S. aureus* USA500 (Diep et al., 2006) was used for construction of gene knockout and complementation strains. *E. coli* DC10B (Monk et al., 2012) was used for shuttle plasmid construction. Luria Broth medium was composed of 1% tryptone (Oxoid), 0.5% yeast extract (Oxoid) and 0.5% NaCl; BM (B-Medium) was composed of 1% tryptone, 0.5% yeast extract, 0.5% glucose, 0.1% K_2_HPO_4_ and 0.5% NaCl; BM and TSB (Tryptic soy broth, Oxoid) were used for *S. aureus* cultivation. Bacterial strains were inoculated in BM, and their growth rate at 37°C was monitored by measuring the OD values at 600 nm. Anhydrotetracycline (ATc) was used for induction of *secY* antisense RNA during gene knockout. Antibiotics were added to medium at the following concentrations: chloramphenicol, 10 μg/ml; ampicillin, 100 μg/ml, levofloxacin, 50 μg/ml.

### Construction of plasmids for homologous recombination and complementary strains

We constructed plasmid pMX10 by replacing *ccdB* element with multiple cloning sites in pKOR1 (Bae and Schneewind, 2006) and used it for construction of gene knock out strains. Primers pMX10-f and pMX10-r were mixed equally to a final concentration of 100 uM, incubated at 72°C for 20 min and slowly cooled to 4°C. The resulting dimers were digested with BamHI and KpnI and ligated to pKOR1 backbone digested with the same restriction enzymes. To construct Δ*ctaB* in USA500, the upstream (us) fragment (about 1000 bp) at the upstream of *ctaB* gene of USA500 strain was amplified with primer ctaB-uf and ctaB-ur, while the downstream (ds) fragment with primers ctaB-df and ctaB-dr. The two fragments were then used as templates for fusion PCR with primer ctaB-uf and ctaB-dr. The final PCR product was digested with KpnI and MluI and then ligated into pMX10. The recombinant plasmids was transformed into USA500 by electroporation and mutants were selected according to the method reported by Bae et al. (Bae and Schneewind, 2006). To construct the complementation strain Δ*ctaB::pRBctaB*, a fragment containing the promoter region and coding sequence of *ctaB* gene was amplified with primers cp-ctaB-f and cp-ctaB-r. The PCR product was digested with EcoRI and BamHI and then ligated into plasmid PRB473. The resulting plasmid was transformed into the Δ*ctaB* mutant via electroporation. The sequences of primers are listed in Table 1.

**Table 1:** **Primers used in this study**.

**Primers**	**Sequence 5′-3′**	**Purpose**
pMX10-f	GGGGTACCGCTAGCCGGCCGGGGCCCACGCGTGAATTCCG	Construction of pMX10
pMX10-r	CGGAATTCACGCGTGGGCCCCGGCCGGCTAGCGGTACCCC	
ctaB-uf	GGGGTACCGCTGTATAACCATAATGAACAGTACG	Construction of Δ*ctaB*
ctaB-ur	CATCCTAACTTAATTAATATCCCCCTCCTTAAATTTGTTC	
ctaB-df	AATTTAAGGAGGGGGATTATTAATTAAGTTAGGATGAAAAATATGGG	
ctaB-dr	CGACGCGTAGAAGTAAGCACTTTAATATCTTTACC	
cp-ctaB-f	CGGAATTCAAAAAGAACTTAATCGTAATGATTTTTTTATTG	Construction of Δ*ctaB::pRBctaB*
cp-ctaB-r	CGGGATCCCTTAATTAATCTAGATCAAAGTAAGTAATGAAAC	
RThld-f	CACTGTGTCGATAATCCATT	Real-time PCR
RThld-r	ATTAAGGAAGGAGTGATTTCAAT	
RTesaB-f	ACTTAGCAGTACCAGCATAT	
RTesaB-r	AATATCTCCATCAGCGATTTG	
RTset18-f	CAGAGCGATTAGCAATGATAA	
RTset18-r	GCGTTCTTGTCTTGTGTTA	
RThtrA-f	TGTGCTATTGAACGATAACG	
RThtrA-r	CTTGCTCTGCTTGATAACTC	
RTarcB2-f	TGAACCTGATGAAGTATGGA	
RTarcB2-r	TGGAAAGATGGTAAGCAATG	
RTsdhA2-f	CAGCAGATTTAGCATTAGCA	
RTsdhA2-r	TACGACCAACCTTATCCATT	
RTnrdE-f	CGATGGTATGGCTATTCCTA	
RTnrdE-r	CGATTGGCATTACAGAACTT	
RTpyrF-f	TAGATGGCGTTGTTTGTTC	
RTpyrF-r	GTAATACGGTGTTGGTCATT	
RTrpmC-f	TTAGAGACTTAACCACTTCAGA	
RTrpmC-r	CTTTCACGAGCAACAGTTT	
RTagrD-f	AACATTGGTAACATCGCAG	
RTagrD-r	GTGTTAATTCTTTTGGTACTTCA	
RTdltA-f	TGGTTCATTCAAGGTCGTA	
RTdltA-r	GCATTGTCCGTAACTTCAG	
RTrrs1-f	GTGCTACAATGGACAATACAA	
RTrrs1-r	ACTACAATCCGAACTGAGAA	

### Detection of pigment production and hemolytic activity

To compare pigment production, USA500 and USA500ΔctaB were dropped onto TSA plates and USA500ΔctaB with pRB473 or pRBctaB on TSA plates with 10 μg/ml chloramphenicol. The plates were incubated at 37°C for 24 h and pictured. For quantitate assay of pigment production, the same strains were cultured in TSB at 37°C for 24 h. For each sample, pigment was extracted with methanol and detected with a parameter (GeneSpec III, Hitachi, Japan), following a previously reported protocol (Morikawa et al., 2001). For hemolytic activity determination, the strains were analyzed by growing the strains on 5% sheep blood agar at 37°C for 48 h. The result represents three independent experiments.

### Mouse infection

The mouse virulence test was performed on Balb/C mice. USA500 and the Δ*ctaB* mutant strains were cultured for 18 h and 1 ml of the each culture was mixed with 2% Cytodex-1 beads by 1:1. The mice were randomized into two groups (5 mice/group). Each mouse was challenged with 200 μl bacterial mixture (each containing approximately 2 × 10^5^ bacterial cells) via injection under skins on the back. After 48 h, the mice were sacrificed and the abscess under skin was homogenized in 2 ml PBS. The samples were diluted and plated on TSA plates at 37°C for 18 h. CFU counting was performed and a Student's *t*-test was used for statistical analysis using Microsoft Excel.

Animal studies on mice were performed according to relevant national and international guidelines (the Regulations for the Administration of Affairs Concerning Experimental Animals, China) and were approved by the Institutional Animal Care and Use Committee (IACUC) of Shanghai Medical College, Fudan University (IACUC Animal Project Number: 20110630). Standard operation procedures were followed to carry out animal experiments in bio-safety level 2 labs.

### Susceptibility testing

The MIC of each antimicrobial compound was determined in triplicate by a conventional broth microdilution technique in TSB medium, following the protocol previously published (Andrews, 2001) and the CLSI guidelines. The MIC was defined as the lowest antibiotic concentration that inhibited visible bacterial growth (also according to OD600 measurements) after 24 h of incubation at 37°C.

### Persister assay

To determine the number of persister cells in exponential phase, cells were grown overnight in 4 ml and were inoculated to 10 ml of fresh medium to an initial OD600 of 0.05. Cultures were shaken for 1.5–2 h (for normally growing cells), until an OD600 of approximately 0.5 was reached. To determine the number of persister cells in stationary phase, overnight cultures (16 or 24 h) were used without dilution.

For heat stress assay, stationary phase cultures were incubated at 57°C for up to 3 h. For oxidative stress assay, stationary phase cultures were diluted by 1:100 in TSB that contained 50 mM hydrogen peroxide (H_2_O_2_) for 4 h. For starvation stress assay, stationary phase cultures were centrifuged, washed and resuspended in 3% NaCl. The survival of bacteria was determined by CFU counting at each hour. All stress assays were conducted using at least three biological replicates.

For antibiotic exposure, 2 ml of the overnight or the exponential phase cultures was transferred to 14 ml culture tubes (Greiner), antimicrobials were added at 100-fold MIC as indicated and the cultures were shaken for 12 h, or for 7 days during long-term experiments. For CFU determination, 100 μl was taken before and during antimicrobial challenge on an hourly basis during the first 8 h and after 24 h, or after 1, 2, 3, 5, 6 days during long-term experiments. Cells were washed in PBS and spotted as 10 μl aliquots of serial dilutions onto TSA plates. Colonies were counted after incubation for 24 h at 37°C. The lower limit of quantification was 100 CFU/ ml. All time-kill experiments were conducted using at least three biological replicates.

### RNA isolation, mRNA enrichment and sequencing

*S. aureus* USA500 parent strain and Δ*ctaB* mutant were cultured for 6 or 24 h as log phase and stationary phase cultures. The cultures were divided into 3 aliquots and treated with RNAprotect (Qiagen) and frozen at −80°C. Total RNA was extracted from bacterial cells using the RNeasy Mini kit (Qiagen) as described (Atshan et al., 2012). The quality of RNA samples was examined with Bioanalyzer 2100 RNA-6000 Nano Kit. To remove 16S and 23S rRNAs, 10 μg of high-quality total RNA was processed using the Ribo-Zero™ Gold Kit before precipitating with ethanol and resuspending into 25 μL of nuclease-free water. The cDNA libraries with 150- to 250-bp multiplexed cDNA were generated from the enriched mRNA samples using the TruSeq Illumina kit (Illumina, San Diego, CA), following instructions from the manufacturer.

Sequencing was performed with HiSeq2500 (Illumina). The Cufflinks suite of tools were used to assess and quantify the total number of reads. With the program Cuffdiff as part of the suite, transcripts were quantified by assessing the total number of reads for the entire transcript. Briefly, reads were mapped to annotated coding sequences (CDSs) from genome of *S. aureus* USA300 TCH1516 strain since USA500 is the progenitor of USA300. The samples to be compared were evaluated for variance and tested for differential expression. Reads' *P*-values were determined, and significance was assessed by conducting Benjamini-Hochberg correction for multiple testing. The transcript sequencing data were submitted to the NCBI Sequence Read Archive, available for access under a RUN number RSS3919726.

### Quantitative real-time PCR

For quantitative Real-time PCR, the same RNA samples were taken from that used for RNA-seq. After reverse transcription with cDNA Synthesis Kit (Bio-Rad Laboratories, Hercules, CA), qRT-PCR was performed using SYBR Green PCR reagents (Takara Biotechnology) to determine the relative expression levels of the target genes with gene-specific primers listed in Table 1. The housekeeping gene *rrs1* (16s RNA) was used as an endogenous control. All qRT-PCR experiments were carried out in triplicate with independent RNA samples and the 2^−ΔΔCT^ method was performed for analysis of relative gene expression data (Livak and Schmittgen, 2001).

### Statistics

The significance of experimental differences in pigment production, hemolytic activity, survival *in vivo* and persister assay was evaluated by unpaired Student's *t*-test.

## Results

### Construction and properties of the *S. aureus ctaB* deletion mutant *ΔctaB*

**Figure 1:**
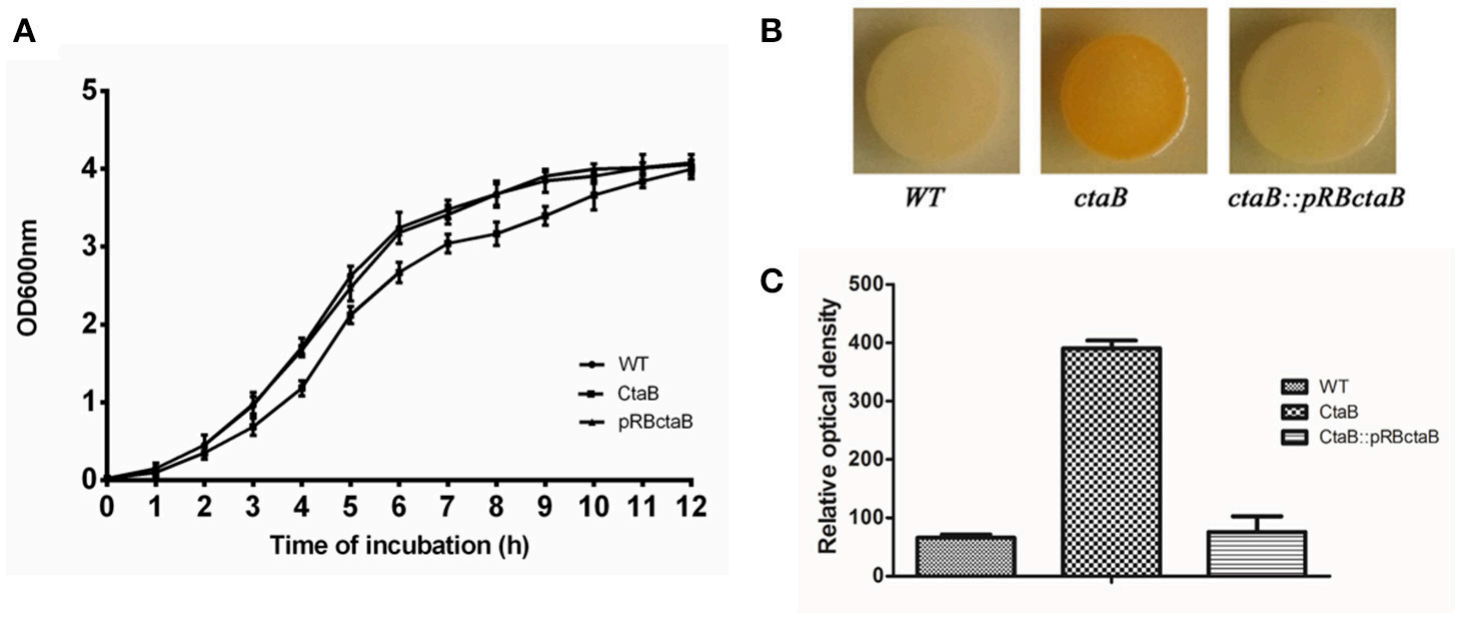
**(A)** Comparison of the growth rate of USA500 and the Δ*ctaB* mutant. A saturated overnight culture of each strain was inoculated in a 12 ml tube and cultured at 37°C. Cultures were monitored by measuring absorption at OD600 each hour. **(B)** Pigmentation display of *S. aureus* strains grown on TSA plates at 37°C for 24 h. **(C)** Measurement of the golden pigment of different strains by methanol extraction. The relative optical density units were detected at 465 nm and normalized to the USA500 strain, which was set at 100. Results are means with standard error (error bars) of three independent experiments.

To investigate the functions of CtaB, we constructed a *ctaB* deletion mutant, Δ*ctaB*, via homologous recombination, as well as made a complemented strain Δ*ctaB::pRBctaB* by inserting *ctaB* with its own promoter into plasmid pRB473. When grown in TSB, the Δ*ctaB* mutant showed a slight growth defect, compared with the parent strain USA500 (Figure 1A). The Δ*ctaB* mutant displayed enhanced golden pigment production when grown on TSA for 24 h compared with the control strain (Figure 1B), and complementation of the Δ*ctaB* mutant reduced pigment production to normal levels (Figure 1B). Quantification of pigment production by extracting carotenoid products confirmed that CtaB depletion afforded enhanced pigmentation than USA500 strain (Figure 1C).

### CtaB affects hemolytic activity and survival *in vivo*

**Figure 2:**
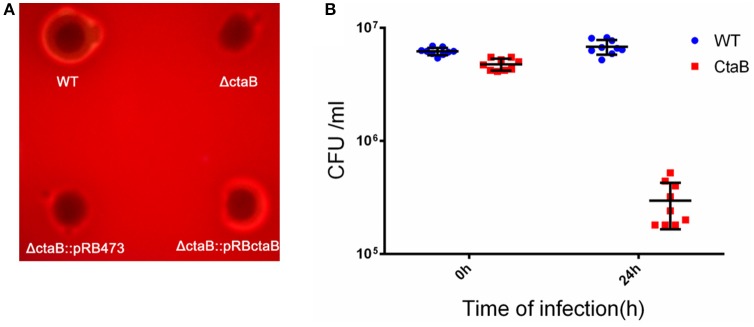
**(A)** Hemolytic activity assay. Overnight cultures of USA500, Δ*ctaB* mutant and complemented strains were spotted (20 μl) on sheep blood agar plates and grown at 37°C for 24 h. The result is representative of triplicate experiments. **(B)** Survival of *S. aureus* strains in a Balb/C challenged by subcutaneous injection. Comparison of CFU counts was performed using the Student's *t*-test. Results are means with standard error (errors bars).

Hemolytic ability is an important aspect of *S. aureus* virulence (Wang and Muir, 2016). We analyzed the level of bacterial growth and hemolysis of the Δ*ctaB* mutant on sheep blood agar plates. While all strains showed similar sized colonies, deletion of *ctaB* generated a strain with reduced hemolytic activity, which could be restored by complementation of the Δ*ctaB* mutant with the wild type *ctaB* gene (Figure 2A).

Having observed that the Δ*ctaB* mutant enhanced pigment production but reduced hemolytic activity, we wondered whether Δ*ctaB* mutant would affect virulence *in vivo*. Skin is one of the most frequently targeted sites for *S. aureus* infection (Liu, 2009). To determine whether CtaB is associated with virulence during *S. aureus* infection, we compared the Δ*ctaB* mutant and the parent strain in a mouse model of skin abscess. Colony counting of bacteria from mouse skin lesions showed that inactivation of *ctaB* attenuated bacterial survival *in vivo*. After 24 h of infection, the CFU counting of USA500 increased from 6.18 ± 0.46E + 6 to 6.79 ± 1.02E + 6. Within contrast, the Δ*ctaB* mutant survived less well with a decrease of CFU, from 4.74 ± 0.57E + 6 to 2.96 ± 1.3E + 6. (Figure 2B).

### CtaB is involved in persister cell formation under stress and antibiotic treatment

**Figure 3:**
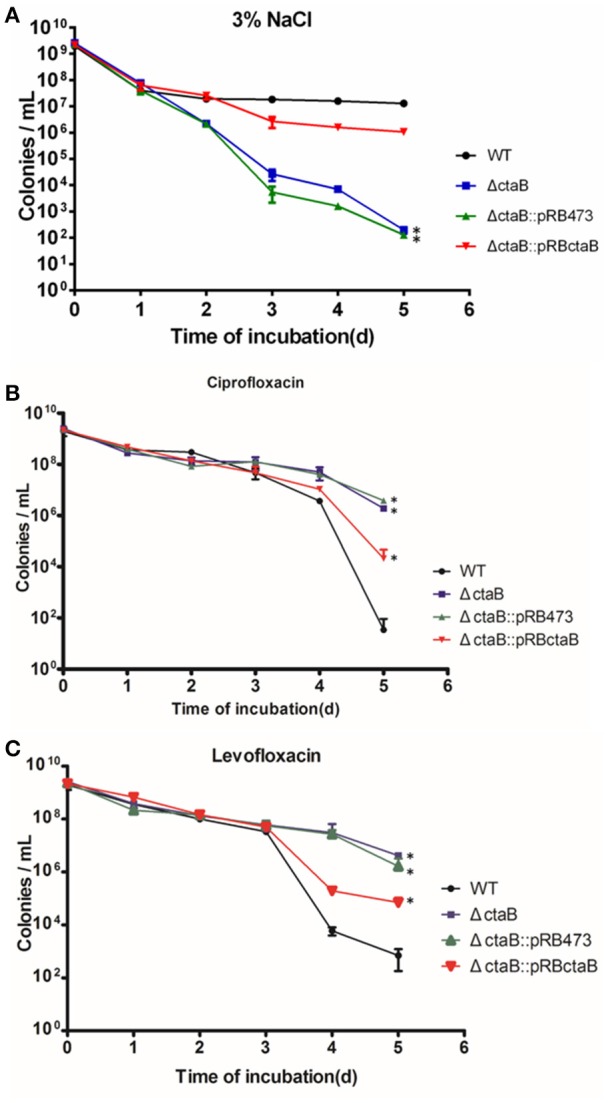
**Time dependent killing of ***S. aureus*** stationary phase bacteria**. **(A)** Effects of starvation (3% NaCl) on survival kinetics of *S. aureus* USA500, Δ*ctaB* mutant and complemented strain. Results are representative of three independent experiments. (**B,C)** Persister assay with antibiotics. Strains were treated with **(B)** 20 μg/ml ciprofloxacin or **(C)** 50 μg/ml levofloxacin for 6 days. The limit of detection was 100 CFU/ml throughout all killing experiments. Results are representative of three independent experiments.

To determine if CtaB is involved in persister formation or survival, we subjected stationary cultures of USA500, Δ*ctaB* and Δ*ctaB::pRBctaB* under stress conditions including heat, oxidative stress, and starvation. The CtaB mutation attenuated the ability of *S. aureus* to survive starvation in 3% NaCl, which provides similar osmotic pressure as TSB, and the impact was reversed by gene complementation (Figure 3A). However, CtaB knockout did not affect survival of *S. aureus* under treatment with heat or H_2_O_2_ (data not shown).

Before persister assay, we measured the antibiotic sensitivity of *ctaB* mutant and found no difference in MIC tests for multiple antibiotics (data not shown). Challenging the stationary phase cultures of USA500, Δ*ctaB* and Δ*ctaB::pRBctaB* with 100 X MIC ciprofloxacin or levofloxacin yielded disparate killing curves. As shown in Figures (3B,C), the surviving ratios of Δ*ctaB* were similar with that of USA500 in the first 3 days, but became higher than the control strain in day four and day five. Meanwhile, complementation with plasmid pRBctaB but not pRB473 partially reversed the augmentation of persister formation caused by deletion of *ctaB* in the last 2 days of treatment. We also tested other antibiotics such as vancomycin, rifampicin, streptomycin, tobramycin, and gentamycin at 100X MIC concentration but found no significant difference in persister formation between the Δ*ctaB* mutant and the parent strain from either exponential phase or stationary phase (data not shown).

### RNA-seq analysis of the *ΔctaB* mutant compared with its parent strain USA500

**Figure 4:**
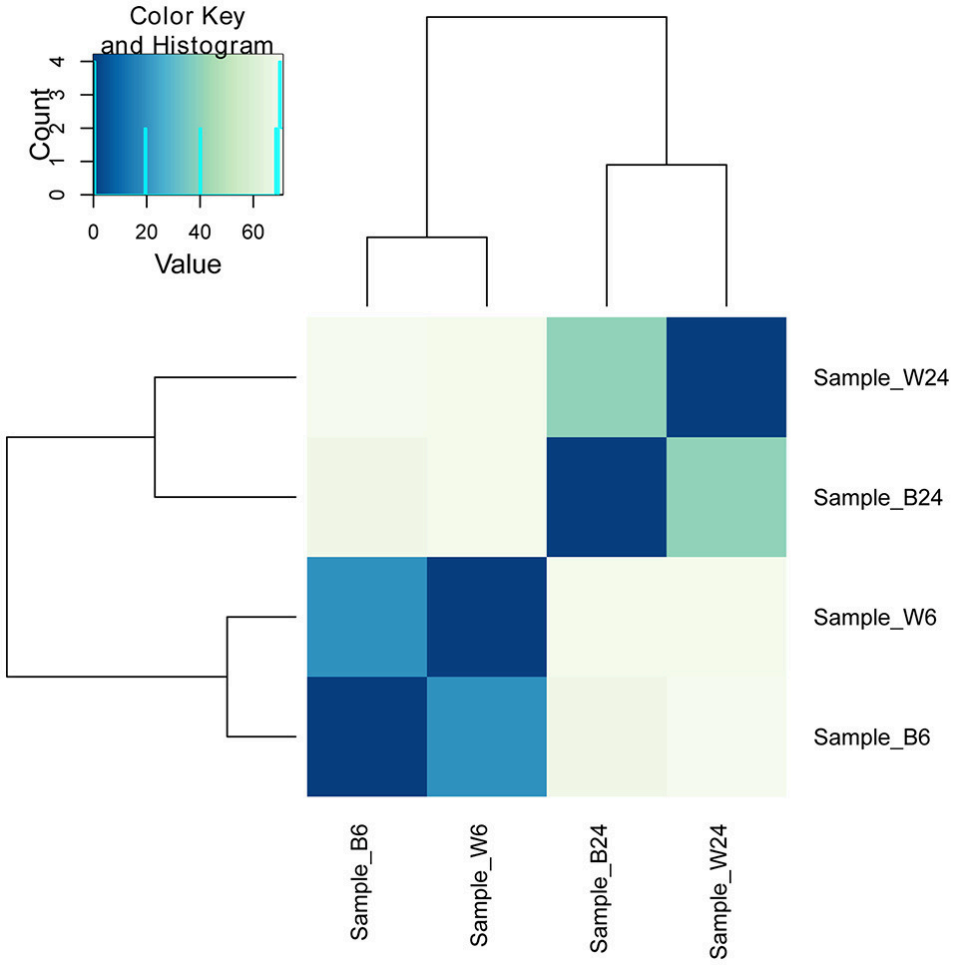
**Clustering of 4 RNA-seq samples**. Sample_W6, USA500 grown to 6 h; Sample_B6, USA500ΔctaB grown to 6 h; Sample_W24, USA500 grown to 24 h; Sample_B24, USA500ΔctaB grown to 24 h. Heatmap shows the Euclidean distances between the samples as calculated from the variance-stabilizing transformation of the count data.

**Figure 5:**
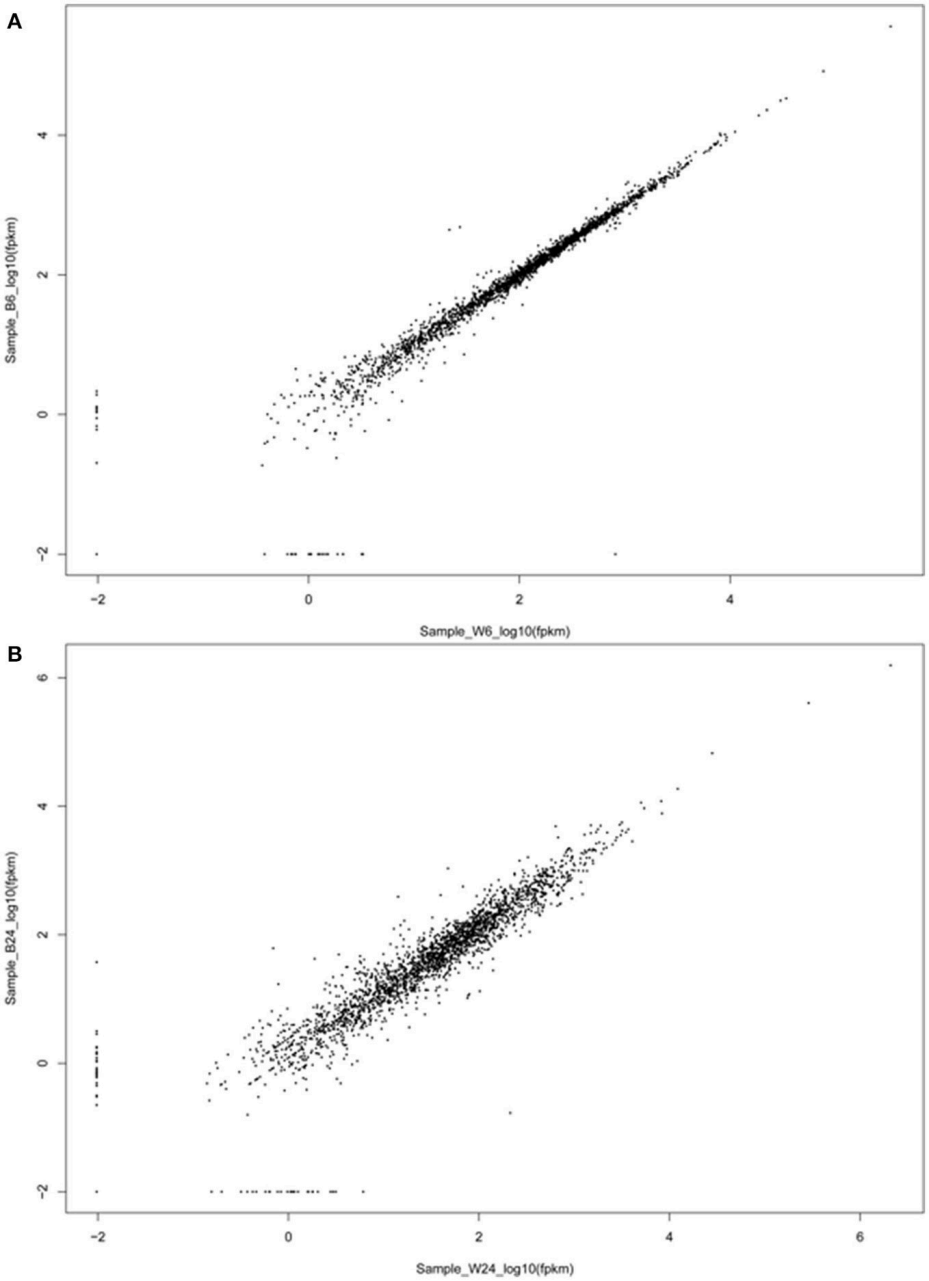
**Scatter plot of the expression levels of pairs of adjacent genes**. The expression levels of two genes located within the same transcript of log phase bacteria **(A)** and stationary phase bacteria **(B)** are plotted in log_10_ scale.

The above results indicate an intriguing and paradoxical role of CtaB in persistence and virulence of *S. aureus*, as its deletion attenuated virulence and survival in 3% NaCl while increasing persister numbers for quinolone antibiotics. To gain insights into the role of CtaB in altered *S. aureus* virulence and persistence, we performed RNA-seq analysis of USA500 and Δ*ctaB* mutant grown for 6 h (log phase) or 24 h (stationary phase) in TSB medium. Based on the results of read counts of all annotated genes, a total of 4 RNA-seq samples were clustered without supervision (Figure 4). The results indicated that the effect of CtaB knockout on the bacterial transcriptome at 24 h were more apparent than that at 6 h (Figures 5A,B).

In log phase cultures, we found 18 genes with significant changes in transcription (cutoff > 2-fold) and *p*-values less than 0.05 between Δ*ctaB* mutant and the parent strain (Table 2). Most strikingly, the virulence gene *hld* was significantly down regulated (0.35, *p* = 8.47E-05) in the Δ*ctaB* mutant. In *S. aureus*, gene *hld* is located inside the coding sequence for small regulatory RNA RNAIII which regulates the expression of many *S. aureus* genes encoding exoproteins and cell-wall-associated proteins. The data indicated that deletion of CtaB could attenuate expression of various virulence genes regulated by RNAIII in log phase. Interestingly, CtaB deletion did not affect expression of the Agr system (encoded by *agrB, agrD, agrC*, and *agrA*), which is the well-known upstream regulator of RNAIII and a virulence factor. The virulence gene *esaB* (Burts et al., 2008; Anderson et al., 2011)was also down regulated. Meanwhile, the virulence genes *set18* and *set19* were up regulated in the Δ*ctaB* mutant. Two hemin ABC transporter super family genes *htrB* and *htrA* were significantly up regulated, as a consequence of lack of heme caused by deletion of CtaB (Table 2).

**Table 2:** **List of genes differentially expressed in USA500 and ΔctaB grown to 6 h**.

**Gene**	**Gene symbol**	**Log_2_ fold change**	***p*-value**	**Description**
USA300HOU_RS*14135*		−2.04	4.4E-05	Hypothetical protein
USA300HOU_RS*09600*		−1.96	1.1E-03	Hypothetical protein
USA300HOU_RS*10850*		−1.83	1.2E-02	Hypothetical bacteriophage protein
USA300HOU_RS *10955*	*hld*	−1.53	8.5E-05	Delta-hemolysin
USA300HOU_RS *03725*		−1.42	9.0E-04	Hypothetical membrane protein
USA300HOU_RS *05170*		−1.24	1.9E-03	Hypothetical protein
USA300HOU_RS *04240*		−1.07	3.7E-02	Hypothetical protein
USA300HOU_RS *10770*		1.02	1.8E-02	Hypothetical protein
USA300HOU_RS *09400*	*ribD*	1.06	9.6E-07	Diaminohydroxyphosphoribosylaminopyrimidine deaminase
USA300HOU_RS *12385*	*ureB*	1.09	1.3E-02	Urease beta subunit
USA300HOU_RS *09390*	*ribA*	1.13	2.0E-07	Bifunctional 3,4-dihydroxy-2-butanone-4-phosphate synthase/GTP cyclohydrolase II
USA300HOU_RS *10800*		1.17	2.8E-03	Hypothetical bacteriophage protein
USA300HOU_RS *09395*	*ribB*	1.32	3.5E-08	Riboflavin synthase alpha subunit
USA300HOU_RS *13155*		1.32	1.6E-02	ABC superfamily ATP binding cassette transporter, ABC/membrane protein
USA300HOU_RS *06710*		1.32	2.3E-02	ABC superfamily ATP binding cassette transporter, membrane protein
USA300HOU_RS *03970*		1.39	4.5E-07	Iron (Fe+3) ABC superfamily ATP binding cassette transporter, membrane protein
USA300HOU_RS *12770*	*htrB*	4.14	1.2E-51	Hemin ABC superfamily ATP binding cassette transporter, ABC protein
USA300HOU_RS *12775*	*htrA*	4.34	1.6E-55	Hemin ABC superfamily ATP binding cassette transporter, membrane protein

**Figure 6:**
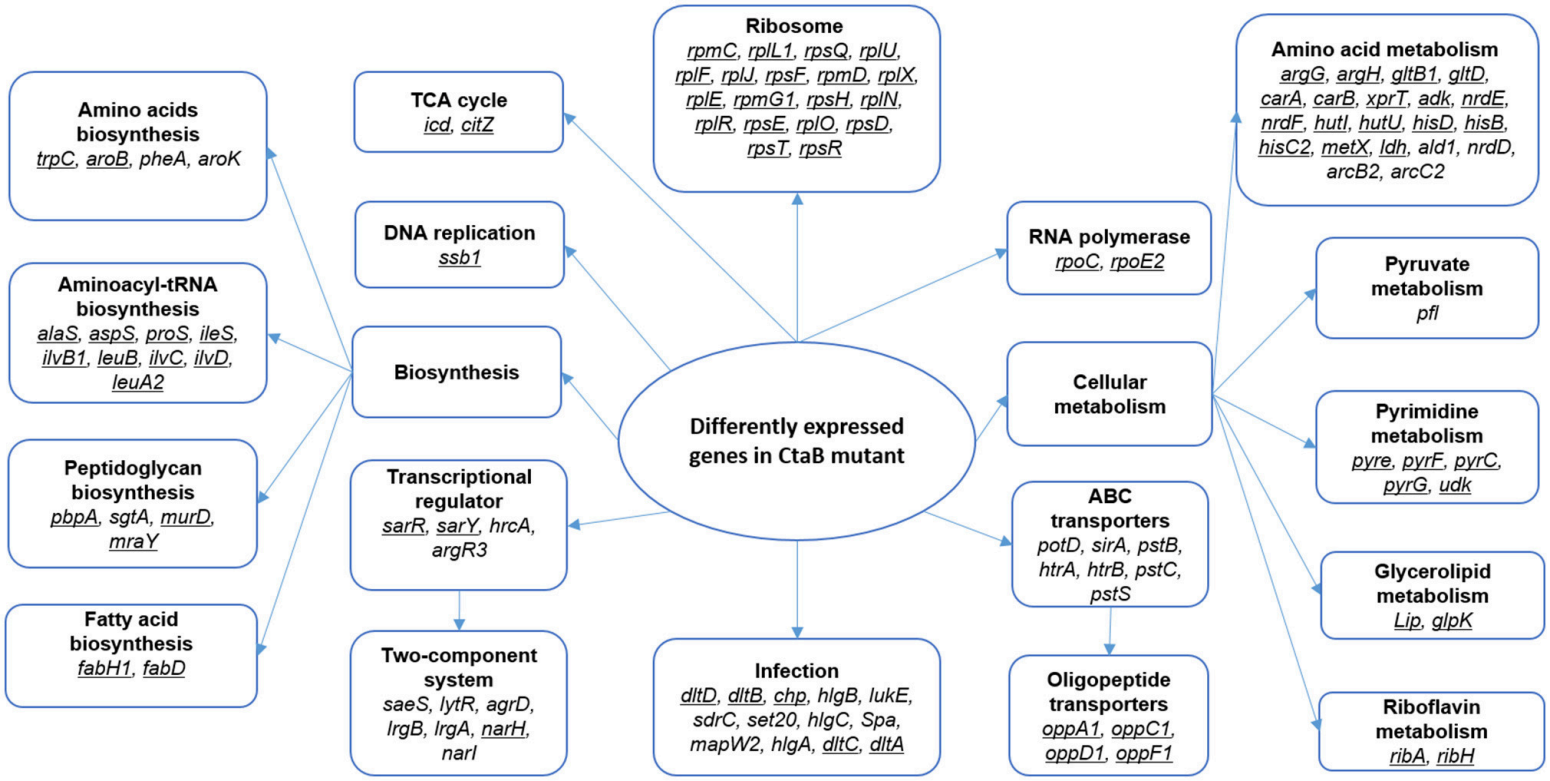
**Proposed pathways affected by depletion of CtaB in stationary phase ***S. aureus*****. Pathways enriched in genes with higher than 2-fold change in CtaB mutant at 24 h are framed and bold. Genes that were down regulated in the CtaB mutant are underlined, while those up regulated not underlined.

For the transcripts at 24 h, 119 genes showed significant changes between Δ*ctaB* mutant and the parent strain (Table 3), indicating that CtaB has a major impact on stationary phase *S. aureus*. The proposed pathways that these genes participate in were analyzed (Figure 6). Nineteen genes encoding ribosome proteins were strongly down regulated, as well as nine genes involved in biosynthesis of Aminoacyl-tRNA. Genes involved in arginine, proline, cysteine, methionine, glycine, serine, threonine, lysine, phenylalanine, tyrosine, tryptophan, valine, leucine, and isoleucine were down regulated in the CtaB mutant. Expression of several ABC transporters was also up regulated, but other transporters such as OppA1, OppB3, OppC1, OppD1, and OppF1, were down regulated. Twenty two genes that encode factors for amino acid metabolism showed difference in expression. Expression of two genes (*arcB2* and *arcC1*) involved in arginine metabolism was up regulated while the others were down regulated. The deletion of CtaB also down regulated genes from pathways involved in purine metabolism, pyrimidine metabolism, and fatty acid biosynthesis. Though out of 17 genes associated with *S. aureus* infection 12 were up regulated, genes in the *dlt* operon (*dltA, dltB, dltC*, and *dltD*) were significantly down regulated (Collins et al., 2002). The CtaB deletion also induced expression of genes of five two-component systems, including PhoPR, LgrAB, SaeRS, and LytSR, indicating that these systems might play a role when *S. aureus* is confronted with lack of heme biosynthesis (Table 3).

**Table 3:** **List of genes differentially expressed in USA500 and ΔctaB grown to 24 h**.

**Gene**	**Gene symbol**	**log_2_ fold change**	***p*-value**	**Description**
USA300HOU_RS01190		−3.37	3.36E-06	Acetyl-CoA C-acetyltransferase
USA300HOU_RS01195		−3.30	7.44E-06	3-hydroxyacyl-CoA dehydrogenase
USA300HOU_RS01200		−3.19	9.02E-06	Acyl-CoA dehydrogenase
USA300HOU_RS01205		−3.16	1.08E-05	Long-chain-fatty-acid–CoA ligase
USA300HOU_RS02265	*cobW1*	−2.78	6.77E-04	Cobalamin (vitamin B12) biosynthesis protein
USA300HOU_RS01210		−2.76	1.58E-04	3-oxoacid CoA-transferase
USA300HOU_RS11065	*ilvB1*	−2.69	1.10E-03	Acetolactate synthase large subunit
USA300HOU_RS02055	*xprT*	−2.66	1.47E-03	Xanthine phosphoribosyltransferase
USA300HOU_RS12265		−2.57	2.28E-03	Hypothetical protein
USA300HOU_RS14500	*lip*	−2.16	2.42E-03	Triacylglycerol lipase
USA300HOU_RS02060	*pbuX*	−2.16	3.25E-03	NCS2 family nucleobase:cation symporter-2
USA300HOU_RS05895		−2.14	1.26E-02	Antibacterial protein
USA300HOU_RS04630	*dltC*	−2.11	7.32E-03	D-alanine–poly(phosphoribitol) ligase
USA300HOU_RS08560		−2.07	1.27E-02	Acetyl-CoA carboxylase biotin carboxyl carrier subunit
USA300HOU_RS12135	*rpmC*	−2.04	3.26E-03	Ribosomal protein L29
USA300HOU_RS11075	*ilvC*	−2.03	1.12E-02	Ketol-acid reductoisomerase
USA300HOU_RS00655		−2.03	3.65E-02	Hypothetical membrane protein
USA300HOU_RS01875		−2.01	4.74E-02	Hypothetical protein
USA300HOU_RS04875	*fabH1*	−1.95	4.11E-03	3-oxoacyl-[acyl-carrier-protein] synthase
USA300HOU_RS02840	*rplL1*	−1.94	5.40E-03	Ribosomal protein L7/L12
USA300HOU_RS12130	*rpsQ*	−1.93	5.02E-03	Ribosomal protein S17
USA300HOU_RS04900	*oppD1*	−1.91	4.92E-03	Oligopeptide ABC superfamily ATP binding cassette transporter, ABC protein
USA300HOU_RS11060	*ilvD*	−1.89	1.79E-02	Dihydroxy-acid dehydratase
USA300HOU_RS07110	*dapB*	−1.84	1.91E-02	Dihydrodipicolinate reductase
USA300HOU_RS13735		−1.82	1.46E-02	Transcriptional regulator
USA300HOU_RS08760	*rplU*	−1.79	1.10E-02	Ribosomal protein L21
USA300HOU_RS07115	*dapD*	−1.75	2.17E-02	2,3,4,5-tetrahydropyridine-2,6-dicarboxylate N-succinyltransferase
USA300HOU_RS07100	*asd*	−1.74	2.52E-02	Aspartate-semialdehyde dehydrogenase
USA300HOU_RS06985	*trpB*	−1.72	4.42E-02	Tryptophan synthase beta subunit
USA300HOU_RS06055	*pyrE*	−1.71	2.74E-02	Orotate phosphoribosyltransferase
USA300HOU_RS06045	*carB*	−1.68	1.67E-02	Carbamoyl-phosphate synthase (glutamine-hydrolyzing), large subunit
USA300HOU_RS01940	*ssb1*	−1.67	1.64E-02	Single-stranded DNA-binding protein
USA300HOU_RS01120	*rplF*	−1.66	1.69E-02	Ribosomal protein L6
USA300HOU_RS02835	*rplJ*	−1.65	1.75E-02	Ribosomal protein L10
USA300HOU_RS02375	*gltB1*	−1.63	2.23E-02	Glutamate synthase (NADPH), large subunit
USA300HOU_RS06050	*pyrF*	−1.62	3.08E-02	Orotidine-5'-phosphate decarboxylase
USA300HOU_RS04905	*oppF1*	−1.58	1.92E-02	Oligopeptide ABC superfamily ATP binding cassette transporter, ABC protein
USA300HOU_RS01935	*rpsF*	−1.56	2.29E-02	Ribosomal protein S6
USA300HOU_RS04620	*dltA*	−1.55	2.45E-02	Long-chain-fatty-acid–CoA ligase
USA300HOU_RS07105	*dapA*	−1.52	4.99E-02	dihydrodipicolinate synthase
USA300HOU_RS04635	*dltD*	−1.51	3.00E-02	D-alanine transfer protein DltD
USA300HOU_RS07190		−1.51	4.86E-02	Nitric-oxide reductase
USA300HOU_RS12610	*hutI*	−1.50	2.99E-02	Imidazolonepropionase
USA300HOU_RS02380	*gltD*	−1.50	4.97E-02	Glutamate synthase (NADPH) small subunit
USA300HOU_RS05945	*murD*	−1.50	2.66E-02	UDP-N-acetylmuramoylalanine–D-glutamate ligase
USA300HOU_RS08555		−1.49	2.77E-02	Biotin carboxylase
USA300HOU_RS12615	*hutU*	−1.49	3.30E-02	Urocanate hydratase
USA300HOU_RS13410		−1.47	3.09E-02	Possible decarboxylase
USA300HOU_RS12085	*rpmD*	−1.46	2.95E-02	Ribosomal protein L30
USA300HOU_RS11770		−1.46	3.46E-02	Hypothetical membrane protein
USA300HOU_RS06545	*glpK*	−1.45	3.79E-02	Glycerol kinase
USA300HOU_RS03955	*nrdF*	−1.45	3.80E-02	Ribonucleoside-diphosphate reductase subunit beta
USA300HOU_RS12120	*rplX*	−1.41	4.01E-02	Ribosomal protein L24
USA300HOU_RS01785	*glpT*	−1.41	3.96E-02	MOP superfamily multidrug/oligosaccharidyl-lipid/polysaccharide flippase transporter
USA300HOU_RS11480	*pyrG*	−1.38	4.72E-02	CTP synthase
USA300HOU_RS06895	*parC*	−1.35	4.42E-02	DNA topoisomerase (ATP-hydrolyzing) ParC
USA300HOU_RS01150	*pfl*	1.33	4.65E-02	Formate C-acetyltransferase
USA300HOU_RS12815		1.37	4.46E-02	Hypothetical lipoprotein
USA300HOU_RS09730		1.39	4.50E-02	hypothetical bacteriophage protein
USA300HOU_RS14200	*nrdD*	1.42	3.47E-02	Anaerobic ribonucleotide reductase large subunit
USA300HOU_RS03820		1.46	3.56E-02	Hypothetical membrane protein
USA300HOU_RS05800		1.47	4.78E-02	Fibrinogen-binding protein
USA300HOU_RS03270		1.49	3.19E-02	Hydrolase
USA300HOU_RS12795		1.51	2.88E-02	Hypothetical membrane protein
USA300HOU_RS03030		1.51	2.58E-02	Hypothetical protein
USA300HOU_RS01345	*scdA*	1.52	2.94E-02	Cell division and morphogenesis protein ScdA
USA300HOU_RS11555		1.54	3.52E-02	Hypothetical protein
USA300HOU_RS12775	*htrA*	1.55	4.66E-02	Hemin ABC superfamily ATP binding cassette transporter, membrane protein
USA300HOU_RS12700		1.56	3.28E-02	Hypothetical protein
USA300HOU_RS03830		1.61	4.27E-02	Hypothetical membrane protein
USA300HOU_RS13375		1.62	2.50E-02	Oligopeptide ABC superfamily ATP binding cassette transporter, binding protein
USA300HOU_RS13655		1.62	3.16E-02	Possible hydrolase
USA300HOU_RS07060	*pstA*	1.63	3.71E-02	Phosphate ABC superfamily ATP binding cassette transporter, membrane protein
USA300HOU_RS08965		1.66	1.63E-02	Sensor histidine kinase
USA300HOU_RS05785		1.69	4.13E-02	Hypothetical protein
USA300HOU_RS13875		1.71	1.13E-02	Hypothetical protein
USA300HOU_RS11550	*dps*	1.71	1.41E-02	Dps family stress protein
USA300HOU_RS14195	*nrdG*	1.72	1.43E-02	Anaerobic ribonucleotide reductase small subunit
USA300HOU_RS01630		1.72	3.52E-02	Acid phosphatase
USA300HOU_RS13840		1.77	2.44E-02	FeoB family ferrous iron (Fe2+) uptake protein
USA300HOU_RS13100	*hlgC*	1.80	1.30E-02	Gamma hemolysin component C
USA300HOU_RS06060		1.83	4.16E-02	Hypothetical protein
USA300HOU_RS01155	*pflA*	1.85	6.42E-03	[Formate-C-acetyltransferase]-activating enzyme
USA300HOU_RS12770	*htrB*	1.86	2.23E-02	Hemin ABC superfamily ATP binding cassette transporter, ABC protein
USA300HOU_RS14125	*fdaB*	1.87	6.17E-03	Fructose-bisphosphate aldolase
USA300HOU_RS13320		1.89	2.63E-02	Hypothetical protein
USA300HOU_RS01220		1.89	1.78E-02	ABC superfamily ATP binding cassette transporter, binding protein
USA300HOU_RS02105	*set3*	1.91	1.38E-02	Staphylococcal exotoxin
USA300HOU_RS01390		1.93	2.02E-02	Hypothetical protein
USA300HOU_RS13085		1.96	4.09E-03	Immunoglobulin G-binding protein SBI
USA300HOU_RS00580	*spa*	1.99	3.51E-03	Immunoglobulin G binding protein A
USA300HOU_RS00555		2.09	3.39E-03	Myosin-cross-reactive antigen
USA300HOU_RS14135		2.14	3.07E-02	Hypothetical protein
USA300HOU_RS10525	*mapW2*	2.14	4.61E-03	Cell surface protein MapW2
USA300HOU_RS12790		2.18	5.84E-03	Hypothetical protein
USA300HOU_RS07065	*pstC*	2.23	4.71E-03	Phosphate ABC superfamily ATP binding cassette transporter, membrane protein
USA300HOU_RS01225		2.26	9.38E-03	Hypothetical protein
USA300HOU_RS01665		2.29	2.74E-03	Possible CNT family concentrative nucleoside transporter
USA300HOU_RS13665		2.29	3.12E-03	MarR family transcriptional regulator
USA300HOU_RS13095	*hlgA*	2.30	8.97E-04	Gamma-hemolysin component A
USA300HOU_RS01655		2.35	2.51E-03	PfkB family carbohydrate kinase
USA300HOU_RS03725		2.41	1.29E-02	Hypothetical membrane protein
USA300HOU_RS04290	*nuc*	2.48	3.60E-03	Micrococcal nuclease
USA300HOU_RS13635		2.50	2.35E-03	ABC superfamily ATP binding cassette transporter, membrane protein
USA300HOU_RS13830	*clp*	2.51	2.93E-04	S14 family endopeptidase Clp
USA300HOU_RS13660		2.53	1.74E-03	Possible lactoylglutatione lyase
USA300HOU_RS01660		2.62	9.10E-04	Hypothetical protein
USA300HOU_RS05775		2.62	2.08E-04	Hypothetical protein
USA300HOU_RS05855		2.80	1.39E-02	Exotoxin
USA300HOU_RS10450		2.80	8.14E-04	Hypothetical protein
USA300HOU_RS07070	*pstS*	2.85	5.80E-05	Phosphate ABC superfamily ATP binding cassette transporter, binding protein
USA300HOU_RS05795		2.90	8.96E-05	Fibrinogen-binding protein
USA300HOU_RS01235	*hmp*	2.96	5.40E-05	Possible nitric oxide dioxygenase
USA300HOU_RS10965	*agrD*	3.45	6.02E-04	Accessory gene regulator protein D
USA300HOU_RS04615		4.03	5.89E-03	Hypothetical protein
USA300HOU_RS13630		4.07	6.62E-06	ABC superfamily ATP binding cassette transporter, ABC protein
USA300HOU_RS01365	*lrgB*	4.08	9.13E-08	Murein hydrolase regulator LrgB
USA300HOU_RS01360	*lrgA*	4.36	1.25E-08	Murein hydrolase regulator LrgA
USA300HOU_RS01230		6.07	1.16E-06	Hypothetical protein

**Figure 7:**
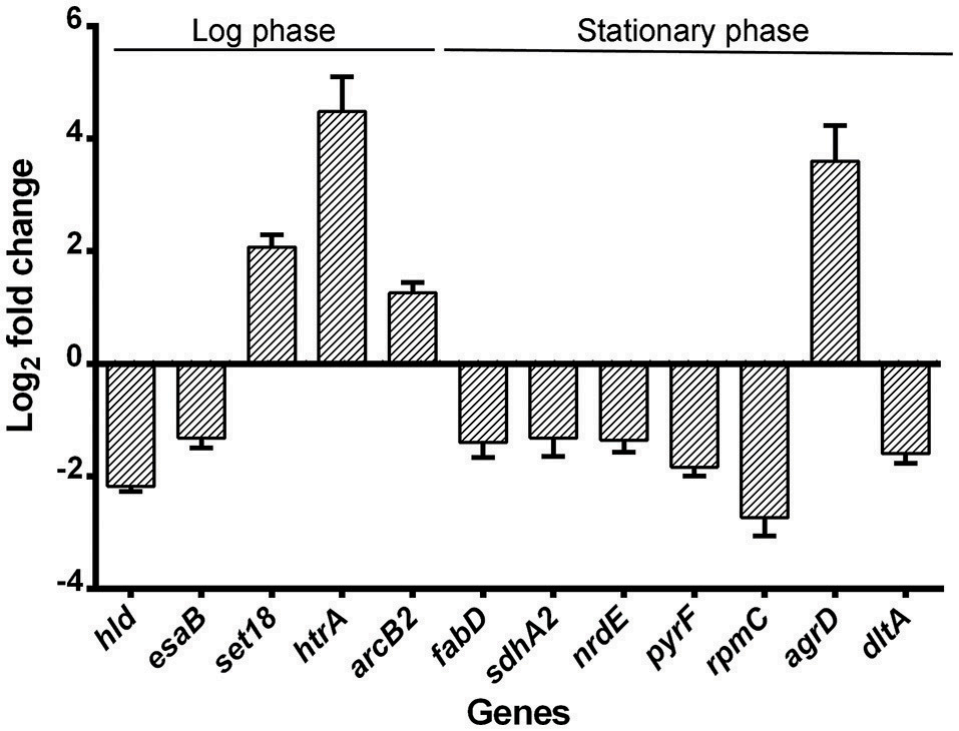
**Validation of RNA-seq by quantitative real-time PCR**. Relative mRNA levels of transcripts corresponding to USA500 and ΔctaB mutant grown to 6 or 24 h were determined. RNA was obtained from the same samples for RNA-seq and experiments were performed in triplicate. Bars show the fold change of Δ*ctaB* mutant vs. USA500 and error bars indicate standard deviations calculated with the 2^−ΔΔCT^ method based on three independent experiments.

Quantitative Real-time PCR was performed to validate the RNA-seq results. Genes were chosen from the list of genes with significant changes of transcription, favoring those associated with virulence and protein production but had a *p*-value < 0.05. All showed similar fold change with those from the RNA-seq results (Figure 7).

## Discussion

The aim of this study is to address the role of CtaB in pigment production, virulence and persister formation in *S. aureus*. We found that deletion of *ctaB* attenuated survival under starvation and virulence in mice but had enhanced pigment production and formation of quinolone tolerant persister cells. Our study is the first to report the complex relationship between heme production, persister formation, and virulence in *S. aureus*.

We have shown that CtaB depletion barely affected growth in rich medium (TSB), but caused faster death under starvation stress (Figure 3A). The result echoes the finding by Clements et al., as CtaA mutation caused growth defect in glucose-limiting chemically defined medium (Clements et al., 1999). Heme production is a key step for cellular aerobic respiration and energy conversion, providing resources for synthesis of heme A-containing terminal oxidases (Svensson and Hederstedt, 1994; Hederstedt et al., 2005). The changes in the respiratory chain by mutation of CtaB could account for the defects. Meanwhile, it is more difficult to explain the enhanced pigment production caused by CtaB depletion. The production of staphyloxanthin, the main pigment of Staphylococci, is mediated by factors encoded by *crtOPQMN*, using FPP as the substrate (Wieland et al., 1994; Pelz et al., 2005). Regulators such as *rsbUVW-sigB* are known to regulate expression of pigment genes in *S. aureus* (Kullik et al., 1998; Giachino et al., 2001). In previous reports, suppression of genes from metabolic pathways (purine biosynthesis, the TCA cycle and oxidative phosphorylation) has also been found to affect pigment production (Lan et al., 2010). We detected the expression of pigment associated genes and found that CtaB deletion did not affect expression of *rsbUVW-sigB, fliA (sigB)* or *crtOPQMN*, while expression of *citZ* in Δ*ctaB* mutant was down regulated (0.45) while *qoxB* was induced (2.09), and the other metabolic genes were not affected (Table 4). FPP is a key intermediate in mevalonate pathway that serves as a substrate of several pathways including synthesis of heme A and staphyloxanthin (Szkopinska and Plochocka, 2005). Since CtaB deletion did not affect pigment production by altering expression of the currently known genes of pigment production pathway, the possibility is worth considering that the absence of competition by heme A production pathway leaves more FPP to staphyloxanthin synthesis pathway, thus enhancing pigment production provided.

**Table 4:** **Transcription change of pigment production associated genes**.

**Genes**	**Fold change (Δ*ctaB* vs. USA500)**	***SD***
*rsbU*	0.90	0.122
*crtM*	1.18	0.143
*qoxB*	2.09	0.276
*citZ*	0.45	0.089
*fliA (sigB)*	0.91	0.129
*purA*	0.56	0.113
USA300HOU_0726 (NWMN_0672)	1.38	0.127
USA300HOU_ (NWMN_1144)	3.54	0.323

From the RNA-seq data, we show the down regulation of multiple virulence genes was caused by CtaB depletion. Despite the depression of global regulatory RNA RNAIII and several classic virulence factors (EsaB, EsaC, EsXB, etc.), DltA-D and most of the amino acid ABC transporters were down regulated. The four proteins (DltA-D) incorporate D-alanine into cell wall polymers during teichoic acid synthesis (Reichmann et al., 2013) and their inactivation has been shown to impact the defense of *S. aureus* against antimicrobial agents (Peschel et al., 1999). Expression of many amino acid transporter genes (*oppA1, oppC1, oppD1*, and *oppF1*) were found down regulated in the CtaB knockout strain. These ABC transporters not only function by obtaining nutrients, but play important roles in adherence and processing of secreting toxins (Podbielski et al., 1996). They also showed up frequently in screening of virulence genes of *S. aureus* with transposon libraries in different animal models (Mei et al., 1997; Coulter et al., 1998; Bae et al., 2004).

Pigment production has been found to enhance fitness and virulence and help the bacteria cope with oxidative stress (Clauditz et al., 2006). However, our results seem to contradict this finding of association of pigment production and virulence as we see enhanced pigment production of CtaB mutant but less virulence. Nevertheless, CtaB deletion had multiple effects on *S. aureus*, despite enhanced pigment production, it caused attenuated hemolytic activity and survival in animal model. It is likely the virulence attenuation of CtaB mutant is combined effect of more important attenuated hemolytic activity over the increased pigment production such that the net outcome is still attenuated virulence despite increased pigment production which is often associated with virulence.

Persister formation is a phenomenon with highly complex mechanisms. Energy production and protein translation are two vital pathways for bacterial survival and reproduction, and it is generally believed that an overall suppression of metabolism and replication is a universal cause for bacterial persister formation (Lewis, 2012; Kwan et al., 2013). In *E. coli*, deficiency of energy production genes such as *sucB* and *ubiF* has been found to decrease persister survival (Ma et al., 2010). It has also been shown in *E. coli* that bacteriostatic antibiotic treatment enhances persister formation via suppression of cellular respiration (Lobritz et al., 2015). In *S. aureus*, a recent study correlated the drop of ATP level to enhanced persister formation in stationary phase (Pontes et al., 2015). CtaB is a key factor in *S. aureus* respiratory chain and energy production. In stationary phase when most glucose is consumed, *S. aureus* turns to utilize amino acids such as arginine and histidine for energy production (Makhlin et al., 2007). We also found that in stationary phase, multiple genes involved in amino acid metabolism (*argG, hutI, hisD*, etc.) were inhibited in Δ*ctaB* strain (Table 3). Based on these findings, CtaB depletion might account for augumented persister formation. However, the correlation between respiration and persister formation is far from unveiled. A counter-example has been provided by Mehmet et al. who reported that inhibition of respiration during stationary phase with KCN reduced persister levels in *E. coli* (Orman and Brynildsen, 2015).

While more work needs to be done to investigate the role of respiratory chain in persister formation, it is also important to further investigate how repression of protein production affects persister formation. The well-understood mechanism of HipAB Toxin-antitoxin system affecting persister formation in *E. coli*, relies on (p)ppGpp to trigger a regulatory cascade involving inorganic polyphosphate (polyP) and Lon, which eventually results in accumulation of (p)ppGpp and persister formation (Rodionov and Ishiguro, 1995; Korch et al., 2003; Germain et al., 2013; Maisonneuve et al., 2013). In our study, CtaB depletion caused strong inhibition of translation by repressing genes involved in multiple aspects of protein production, including amino acid transport (*oppA1, oppC1, oppD1*, etc.), amino acid synthesis (trpC, aroB,), aminoacyl-tRNA biosynthesis (*aspS, alaS, ileS*, etc.) and ribosome proteins (*rpmC, rplF, rpsE*, etc.) (Table 3; Figure 6).

It is generally assumed that elevated persistence is associated with better survival and therefore higher virulence in animal models. However, our observation that mutation of CtaB caused attenuated virulence but elevated persister formation, seems paradoxical. Indeed, many infectious diseases are difficult to be treated with antibiotics due to persisters but not resistance (Mulcahy et al., 2010; Welsh et al., 2011). However, we propose that in most cases with MRSA infection the role of virulence is greater than persister formation because: the proportion of persisters is generally small; after antibiotics kill the majority of infecting population of bacteria, the host immune system generally eliminates persisters non-selectively. Nevertheless, the importance of persister formation by MRSA should not be neglected. Future studies are needed to better understand the roles virulence and persistence play in *S. aureus* infection.

In our study, the CtaB mutant only showed elevated persister formation with levofloxacin and ciprofloxacin but no other antibiotics. This may be attributed to the multi-drug resistance of the MRSA strain, which may have masked the defect in persistence to other antibiotics. The difference in persister formation between MRSA and methicillin-sensitive *S. aureus* (MSSA) is worth further investigation.

Our study suggests the importance of heme synthesis in virulence and persister formation of *S. aureus* and provide new insights into the role of CtaB in bacterial respiration in *S. aureus* virulence and persistence. However, one limitation of the study is that we have not dealt specifically with the metabolic aspects of CtaB mutation, such as the efficiency of the respiratory chain in the mutant and possible changes in components of the TCA cycle as well as comparing the phenotypes of *S. aureus* and the *ctaB* mutant in anaerobic conditions. Future studies are needed to address these issues and better understand the relationship between *S. aureus* respiration and virulence and persistence.

## Author contributions

YZ, TX, and WZ designed the work and revised the manuscript; TX, JH, JZ, JC, and NW completed all the experiments; TX and JH performed the statistically analysis and made the figures; TX and YZ wrote the manuscript.

### Conflict of interest statement

The authors declare that the research was conducted in the absence of any commercial or financial relationships that could be construed as a potential conflict of interest.

## Abbreviations

MRSA: methicillin resistant *Staphylococcus aureus*
FPP: farnesyl diphosphate
SCV: small colony variants
MIC: minimum inhibitory concentration.
